# Small reductions in cargo vessel speed substantially reduce noise impacts to marine mammals

**DOI:** 10.1126/sciadv.adf2987

**Published:** 2023-06-21

**Authors:** Charlotte R. Findlay, Laia Rojano-Doñate, Jakob Tougaard, Mark P. Johnson, Peter Teglberg Madsen

**Affiliations:** ^1^ Zoophysiology, Department of Biology, Aarhus University, Aarhus 8000, Denmark.; ^2^ Department of Ecoscience, Aarhus University, Aarhus 8000, Denmark.

## Abstract

Global reductions in the underwater radiated noise levels from cargo vessels are needed to reduce increasing cumulative impacts to marine wildlife. We use a vessel exposure simulation model to examine how reducing vessel source levels through slowdowns and technological modifications can lessen impacts on marine mammals. We show that the area exposed to ship noise reduces markedly with moderate source-level reductions that can be readily achieved with small reductions in speed. Moreover, slowdowns reduce all impacts to marine mammals despite the longer time that a slower vessel takes to pass an animal. We conclude that cumulative noise impacts from the global fleet can be reduced immediately by slowdowns. This solution requires no modification to ships and is scalable from local speed reductions in sensitive areas to ocean basins. Speed reductions can be supplemented by routing vessels away from critical habitats and by technological modifications to reduce vessel noise.

## Teaser

Cumulative noise impacts on marine mammals from the global cargo vessel fleet can be reduced immediately by slowing down.

## INTRODUCTION

More than 80% of international trade is conducted by motorized vessels at sea (*1*), making shipping the most widespread human noise source in marine ecosystems (*1*–*4*). Mounting evidence shows that vessel noise has detrimental effects on fish, invertebrates, and marine mammals that may culminate in population-level consequences (*5*, *6*).

Marine mammals are of particular concern as many species rely on hearing as their primary sense for foraging, orienting, detecting predators, and communicating (*7*). Noise from shipping can affect marine mammals in several ways. Underwater radiated noise from vessels can directly affect behavior (*5*) via disruption to foraging (*8*, *9*), nursing, or resting (*10*, *11*) and may mask acoustic cues important for social interactions and migration (*12*). Although studies often use the maximum level of vessel noise exposures as a proxy for severity, behavioral responses may also be mediated by the rate of change in received level (*13*), an effect that we term “acoustic looming” (see glossary of terms). Acoustic looming is analogous to the perception of predation risk by animals (*14*), whereby a fast-increasing received level, e.g., due to a rapidly approaching vessel, could provoke a stronger or more urgent response, thereby serving as a sensory cue for the immediacy of the threat (*15*). However, even lower and slower-changing noise levels that are less likely to invoke strong behavioral responses may still mask acoustic communication between conspecifics, prey detection, and the reception of other sounds of importance such as from predators (*16*, *17*). In this case, the duration of time that vessel noise elevates the ambient noise level is a relevant measure of impact. Thus, there are multiple ways in which vessel noise can potentially affect marine mammals and, consequently, a variety of measures of noise exposure. Although work is ongoing to develop tools for mapping vessel noise impacts to marine species (*18*), the threshold of response for each measure likely varies with context, habitats, and species, complicating both the assessment of impacts and efforts to mitigate vessel noise.

Concern for the increasing and widespread impacts to marine wildlife from vessel noise has led regulators to take measures to reduce underwater radiated noise (*19*–*23*). Three general approaches have been proposed: (i) reducing the noise produced through vessel slowdowns (*24*–*26*), (ii) technological modifications to reduce radiated noise levels (*27*, *28*), and (iii) reducing the received noise through increasing the separation between vessels and animals in both space and time (*29*). Although increasing the distance between vessels and animals by moving shipping lanes can be effective at reducing vessel noise (*26*), it requires considerable understanding of species habitat use and can require large detours to reduce noise levels sufficiently, making it infeasible in restricted waterways (channels, rivers, etc.). Therefore, increasing focus has been placed on measures to reduce vessel source levels through slowdowns and technological changes (*24*–*28*).

Although reducing the radiated noise from vessels, howsoever achieved, must generally reduce the risk of disturbance, the efficacy of these mitigation measures has rarely been quantified. Here, we use a modeling approach to explore how changes in vessel speed, distance to vessel, and vessel design affect three proxies for impact: maximum received level, acoustic looming, and exposure duration. We examine how the area and thereby, on average, the number of animals exposed to vessel noise changes as a function of vessel speed and noise-reducing technological improvements and how increasing the distance to animals affects the proxies for impact. We also test the frequently invoked concern about slowdowns that the resulting reduction in source level is offset by the longer time the ship spends within the habitat (*30*, *31*).

## RESULTS AND DISCUSSION

### Vessel noise substantially alters ambient soundscapes

**Fig. 1.**
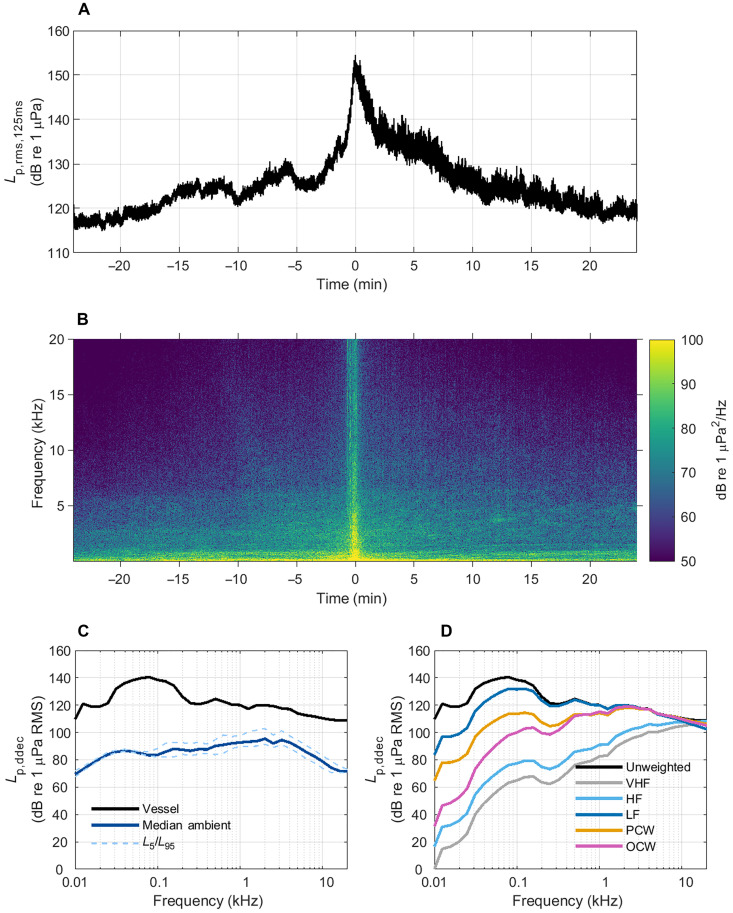
Cargo vessel noise alters local ambient soundscape. (**A**) Broadband received level (*L*_p,125ms_) (*57*) of a container ship passing a static passive acoustic recorder (Kattegat, Denmark, 56° 55.139′N, 11° 45.492′E; depth, 35 m; DSG-ST Ocean Acoustic Recorder, Loggerhead Instruments, USA, 96-kHz sampling rate) at a speed of 15.7 knots. (**B**) Spectrogram of the same vessel pass limited to an upper frequency of 20 kHz (32,768-point Fast Fourier Transform size, Hann window, 50% overlap). Time 0 refers to the closest point of approach (CPA). (**C**) Received decidecade levels (average *L*_p,ddec_; dB re 1 μPa; ± 30 s around time 0) of the vessel passage, and the median and exceedance levels (*L*_5_ and *L*_95_; the levels exceeded 5 and 95% of the time, respectively) of the ambient noise recorded in the same area over a 2-hour period (Kattegat, Denmark, 56° 54.099′N, 11° 38.891′E; depth, 18 m; DSG-ST Ocean Acoustic Recorder, Loggerhead Instruments, USA). (**D**) Decidecade spectra weighted by the estimated hearing response of different marine mammal hearing groups (VHF, very high-frequency cetacean; HF, high-frequency cetacean; LF, low-frequency cetacean; PCW, phocid carnivore in water; and OCW, other carnivores in water) (*37*).

Vessel noise from cargo ships substantially alters ambient soundscapes especially at low frequencies (*32*–*34*). Figure 1A shows an example of the noise produced by a cargo vessel (a container ship) passing within 40 m of a static passive acoustic recorder in 35-m-deep water. During the passage, broadband received sound pressure levels (*L*_p,rms,125ms_) increase to a maximum of 155 dB re 1 μPa at the time of closest approach (Fig. 1A) and elevate the ambient noise levels for over 40 min. Analysis of the source levels of all cargo vessel classes from the Haro Strait, Canada, reported the greatest mean monopole source levels (>170 dB re 1 μPa m) in decidecade frequency bands (*L*_p,ddec_) below 100 Hz (*25*). In Fig. 1B, most of the vessel noise power occurs at frequencies above 20 Hz with a maximum level of 141 dB re 1 μPa in the 80-Hz decidecade band (*35*), which exceeds the median ambient decidecade level by 57 dB (Fig. 1C). However, as shown in Fig. 1B, cargo vessels also produce substantial noise at higher frequencies (*36*), and the effect of this noise on soundscapes is accentuated by the typically lower-amplitude, high-frequency ambient noise. Consequently, motorized vessels can alter the ambient soundscape across a broad frequency range for protracted periods. The impact of this on an animal depends on the source level of the vessel, the ambient noise levels, the receiver distance, and the animal’s hearing acuity.

Different species of marine mammals perceive the loudness of the same ship differently. These differences in audibility can be accounted for by applying frequency weighting functions to the ship noise spectrum matched to different marine mammal functional hearing groups (Fig. 1D) (*37*). The weighting functions are approximations to the audiograms for each hearing group. For example, low-frequency cetaceans, phocid carnivores in water, and other carnivores in water are expected to perceive ship noise as louder at lower frequencies (<1 kHz), whereas high-frequency and very high-frequency cetaceans perceive the higher frequencies better (Fig. 1D). Because of the differences in hearing abilities, the response to the same vessel passage is expected to vary between marine mammal groups. However, regardless of the species of interest, approaches that reduce the broadband underwater radiated noise from vessels will likely benefit all marine mammal groups and other sound sensitive marine species (fish, invertebrates, etc.).

### Moderate source-level reductions greatly reduce the area exposed to vessel noise

**Fig. 2.**
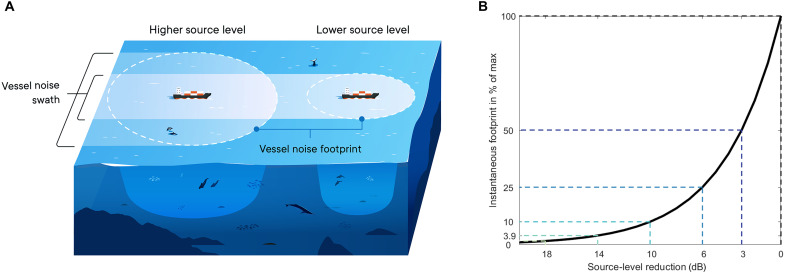
Source-level reductions reduce the area and volume of water exposed to vessel noise. (**A**) Illustration highlighting the spatial extent of the noise radiated from two otherwise identical ships with different source levels (difference of 6 dB). The spatial extent can be expressed not only as the instantaneous surface footprint and the associated exposed volume underneath the vessel but also as the swath created by the vessel as it moves through the habitat. Illustration by Amy Dozier (MaREI, the SFI Research Centre for Energy, Climate and Marine Environment Research Institute, University College Cork). (**B**) Instantaneous footprint area reduction (as percent of max) as a function of source-level reduction (decibels).

The potential for shipping noise to affect marine wildlife can be quantified by the area of habitat degraded by the presence of a ship (*38*, *39*). Figure 2A illustrates two otherwise identical vessels, one with a source level of 6 dB lower than the other as a result, for example, of a difference in speed and/or differences in construction or propulsion technology.

Although vessel noise propagates in three dimensions, the acoustic footprint at the surface is a useful proxy for the region affected by noise. More precisely, we define the instantaneous acoustic footprint as the area exposed to underwater radiated noise exceeding the ambient noise level at a point in time (Eqs. 1 to 4). To exemplify this, we selected a reference scenario of a 295-m container ship traveling at a high speed of 20 knots. Compared to this reference ship, a source-level reduction of 6 dB will result in a 50% reduction in the swath (the strip ensonified as the ship moves through the habitat) and a 75% reduction in the instantaneous acoustic footprint, under a spherical spreading loss propagation model. Assuming that marine mammals are distributed evenly in the habitat, this also means an average 75% reduction in the number of marine mammals affected by the noise at any given time (Fig. 2, A and B). This reduction increases markedly as source levels are reduced further, with a 10-dB reduction resulting in a 90% decrease of the instantaneous acoustic footprint. Consequently, even a moderate reduction in a vessel’s source-level results in a marked reduction in the acoustic footprint of the vessel, owing to the inverse square law of spherical spreading (Fig. 2B). This reduction in source-level results in far fewer exposed animals, no matter whether behavioral responses or masking are considered.

In a real habitat, the extent to which vessel noise propagates, and thereby the extent of the acoustic footprint, depends on local environmental conditions (sound speed profile, sediment, depth, etc.), as well as the spectrum and directionality of the ship source itself (*27*, *40*, *41*). In shallow-water environments, the attenuation of sound with distance is complex and falls somewhere between the spherical spreading [20log_10_(distance)] that we have assumed and cylindrical spreading [10log_10_(distance)]. Range-dependent propagation models matched to the environment (*42*) provide more accurate quantification of the acoustic footprint of a vessel. However, lower spreading loss relative to spherical spreading only increases the beneficial effects of source-level reductions highlighted here. For example, a 6-dB reduction in source level reduces the area exposed to a given sound level by 75% under spherical spreading but by 94% under cylindrical spreading. Therefore, the choice of a spherical spreading loss model is conservative in the sense that a reduction in ship source level will likely reduce the relative area over which marine mammals are affected by even more than our estimates suggest, especially in shallow-water environments (Fig. 2B).

### Moderate slowdowns strongly reduce vessel source levels

**Fig. 3.**
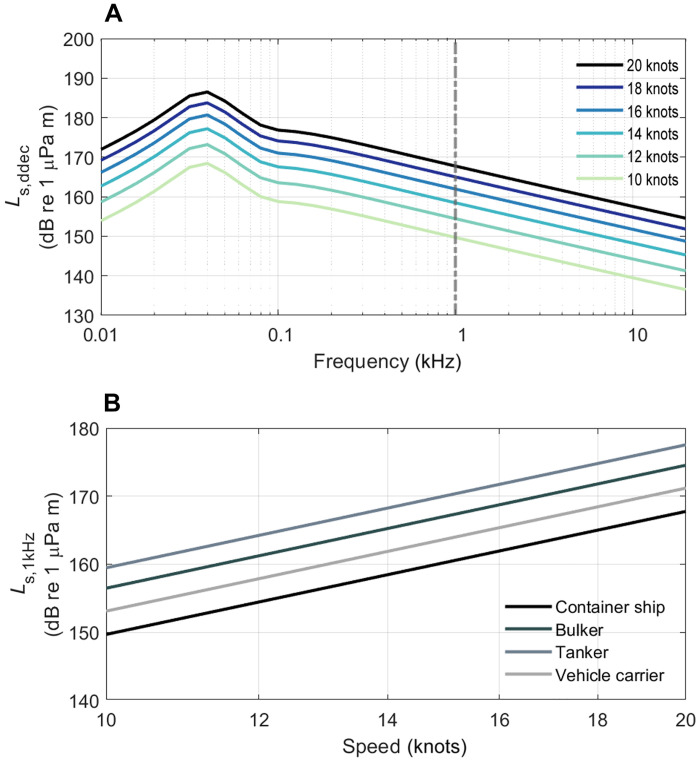
Slowdowns are effective at reducing cargo vessel source levels. (**A**) Predicted decidecade source levels (*L*_s,ddec_; dB re 1 μPa m) for a 295-m container ship with a fixed pitch propeller traveling at high speed (20 knots) and then 10 to 50% slower. Gray dashed line indicates the 1-kHz source level that is audible to all marine mammal functional hearing groups (*7*, *37*). Values used to construct the curves, as well as auditory frequency weighted levels for different groups of marine mammals, can be found in table S1. (**B**) Predicted source levels in the 1-kHz decidecade (*L*_s,1kHz_) of different cargo vessel classes (container ship, bulker, tanker, and vehicle carrier) of 295-m-length traveling at different speeds.

To determine the efficacy of slowdowns, we quantified the change in source levels as a function of speed using the ship source spectrum model by MacGillivray and de Jong (*43*). This model provides average source levels and frequency spectra for different ship classes as a function of ship length and speed (Eq. 6). The model is based on extensive empirical observations that radiated noise is proportional to the sixth power of speed for fixed pitch propeller cargo vessels operating above the cavitation inception speed (typically, 9 to 14 knots) (Eq. 8 and Fig. 3, A and B) (*32*, *44*). This means that even small slowdowns result in considerably lower source levels (Fig. 3). Specifically, a 20% reduction in speed decreases the predicted mean source level by 6 dB and a 50% speed reduction drops mean source levels by 18 dB (Fig. 3A). This level reduction is approximately equal across frequency, meaning that a 20% reduction in speed reduces the sound output from a vessel at all frequencies by 6 dB. Individual vessels and different vessel classes can vary widely in their source level due to differences in design and maintenance, but, as shown in Fig. 3, running at a lower speed is a broadly effective way to reduce the source level of most vessels relative to their usual operating speed (*43*). An exception is vessels with controllable pitch propellers (e.g., tugs and ferries), which can produce more noise during low-speed maneuvering. However, this propeller type makes up only a small proportion of the global fleet (*19*, *45*), and, during higher-speed transits, these vessels generally perform like fixed-pitch vessels.

Slowdowns are increasingly being trialed to reduce vessel noise and its impacts to marine life. A voluntary vessel slowdown to 11 knots within Haro Strait, Canada, as part of the Vancouver Fraser Port Authority Enhancing Cetacean Habitat and Observation (ECHO) program resulted in an 11.5-dB reduction in the mean broadband monopole source levels of container ships (*25*). Speed reductions are already used for economic reasons in commercial shipping when overcapacity in the market exists and when transport costs can be lowered by traveling slower than the vessel design speed (*46*). Faber *et al.* (*47*) argued that a 50% reduction in speed compared to “business as usual” may be difficult to achieve due to time and economic constraints. However, they suggest that a 10 to 30% reduction (giving a predicted 3 to 10-dB source-level reduction) is feasible for most ship types, although this may require an increase in fleet capacity to maintain cargo throughput (*47*). In addition to reducing noise outputs, slowdowns can improve fuel consumption by cargo vessels (*28*, *45*, *46*, *48*), saving both money and greenhouse gas emissions, thereby helping the shipping industry to meet climate targets set by the International Maritime Organization (*45*, *47*–*50*). A moderate reduction in speed is, therefore, an effective, immediate, and readily implemented approach for reducing the source levels of fixed pitch propeller cargo vessels, with positive economic and climatic synergies (*45*, *46*). However, given that slower vessels take longer to pass an animal, do reductions in speed translate unequivocally in reduced impacts on marine mammals or is it a zero-sum game (*25*, *26*, *30*, *31*)?

### Slowdowns reduce noise impacts to marine mammals

**Fig. 4.**
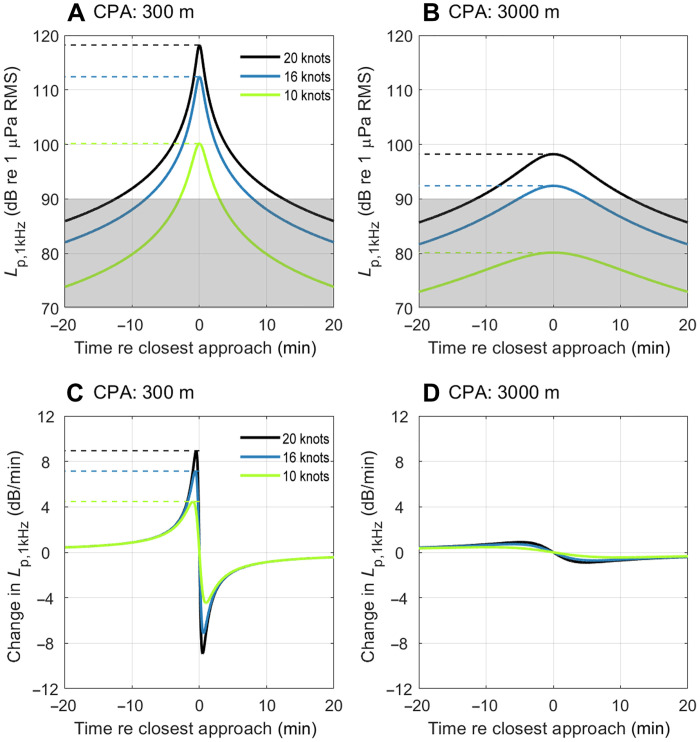
All noise impacts to marine mammals decrease if vessels are slowed down or are at greater distances from the receiver. (**A**) Received level in 1-kHz decidecade band (*L*_p,1kHz_) predicted for a 295-m cargo vessel passing 300 m from an animal. (**B**) *L*_p,1kHz_ for the same vessel predicted for an animal at 3000 m. (**C**) Acoustic looming, i.e., the change in *L*_p,1kHz_ predicted for an animal at 300 m and (**D**) at 3000 m. Colored lines extending to the *y* axis in (A) to (C) indicate maximum *L*_p,1kHz_ and peak change in *L*_p,1kHz_ for vessels traveling at different speeds [high speed, 20% slowdown (16 knots), and 50% slowdown (10 knots)], and gray transparent areas in (A) and (B) indicates values below the ambient noise level (*L*_p,amb,1kHz_; taken as 90 dB re 1 μPa). Values for maxima on the curves can be found in table S2.

To assess the effectiveness of the slowdown approach at reducing noise impacts to marine mammals, we modeled three speed scenarios (high speed and a 20 and 50% slowdown), assuming that vessels were passing a stationary animal at either 300- or 3000-m closest distance in an otherwise quiet habitat. When animals were exposed to slower vessels, all three noise impact proxies—maximum received level, exposure duration, and peak acoustic looming—were reduced (Fig. 4). For example, if a vessel passing 300 m from an animal is slowed down by 20% (giving a predicted 6-dB source-level reduction), then the maximum received level of the vessel decreases from 118 to 112 dB re 1 μPa (Fig. 4A), the exposure duration reduces from 25 to 16 min (assuming an ambient noise level of 90 dB re 1 μPa; Fig. 4, A and B, and table S2), and peak acoustic looming drops from 8.9 to 7.1 dB/min (Fig. 4, C and D). Although exposure duration depends on the prevailing ambient noise level, the relative change in exposure duration due to slowing down is independent of the ambient noise level provided that the vessel pass is reasonably long (see Materials and Methods). For example, at a lower ambient noise level of 70 dB re 1 μPa, the exposure duration shows the same 36% reduction for a 20% slowdown (a decrease from 250 to 160 min). However, at high ambient noise levels for which the exposure durations are already short, the relative gains from speed reduction become even more pronounced (e.g., at 110 dB re 1 μPa, a slowdown of 20% reduces exposure duration by 60%, from 2.3 to 1 min). Thus, given the strong relationship between speed and source levels, even a moderate vessel slowdown of 20% reduces all potential noise impacts to marine mammals at a given distance. Moreover, this speed reduction effectively halves the vessel noise swath, reducing the average number of animals exposed by 50%. Even greater impact reductions accrue with more substantial slowdowns; for example, a 50% speed reduction could be used within marine protected areas and other sensitive habitats to reduce the instantaneous noise footprint of vessels by 98.5%, substantially decreasing noise impacts to marine mammals.

There is growing empirical evidence that slowdowns are an effective mitigation strategy. Voluntary slowdown within key habitats for critically endangered Southern Resident killer whales (*Orcinus orca*) and associated noise reductions during the ECHO program resulted in a 22% decrease in the potential lost foraging time (*24*). Slowdowns have the added benefit of reducing fatal ship strikes (*29*, *51*). However, concerns have been raised that slowdowns, by prolonging the period over which animals are exposed to vessel noise, could end up being a “zero-sum game” (*30*, *31*); animals are exposed less but for longer durations, potentially weakening the benefit of this mitigation strategy. However, our results show that this is not the case: Vessel slowdown also reduces the overall exposure time during which an animal may be affected by noise. Compared to a fast vessel passage (20 knots), a slowdown of 50% (i.e., to 10 knots) reduces the time during which an animal is exposed to ship noise above ambient (assumed to be 90 dB re 1 μPa Root Mean Square (RMS) by 76% for all frequencies at the closest approach distance of 300 m. For a 3000-m closest approach distance, noise from the slower vessel may never exceed ambient (Fig. 4, A and B), making masking and behavioral disturbance unlikely. In support of this, recent field measurements of cargo vessels from the Santa Barbara Channel, USA, showed that faster vessel transits resulted in noise remaining more than 15 dB above the ambient for longer compared to slower transits, which authors concluded was due to the strong relationship between vessel source levels and speed (*52*). Consequently, vessel slowdowns reduce both received noise levels and exposure duration despite the increased time the vessel spends within a habitat.

One negative consequence of slowing down is the possible need for more ships if there is no spare capacity in the fleet (*47*). Some types of cargo ships form part of a supply chain that must maintain a steady throughput of goods and services [e.g., in 2021 around 1.8 billion metric tons of crude oil were transported via maritime routes (*53*)]. To maintain this stream of cargo with slower ships, more ships may be needed, in inverse proportion to the slowdown. However, the increase in number of ships is counterbalanced by the fact that slower ships call into port less often and the net benefit of slowing down thus remains unaffected by the increase in ship number.

### Increasing distance and slowing down reduce impacts to marine mammals

Increasing the distance between vessels and marine mammals alone or in combination with slowing down reduces all measures of noise impacts, albeit to varying degrees (Fig. 4). For example, increasing the closest approach distance from 300 to 3000 m alone reduces the maximum received level, exposure duration, and acoustic looming of a vessel traveling at 20 knots (118 to 98 dB re 1 μPa, 25 to 23 min, and 8.9 to 0.9 dB/min). While the effect on maximum received level and looming is strong, exposure duration reduces weakly with minimum separation distance because the vessel may still be audible and, therefore, mask an animal’s hearing, at many times this distance. A stronger reduction across all measures is achieved if the vessel is also slowed by 20% (92 dB re 1 μPa, 10.4 min, and 0.7 dB/min; Fig. 4B). Thus, there is a synergistic effect in combining mitigation strategies.

Speed restrictions and the rerouting of shipping lanes away from important habitats have been effective at reducing noise within frequencies used for calls and echolocation in Southern Resident killer whales (*26*). These steps have also reduced ship-strike deaths in North Atlantic right whales (*Eubalaena glacialis*) (*29*, *51*). The efficacy of rerouting as a noise mitigation strategy depends on the local sound propagation conditions, geographic and bathymetric constraints, and the degree to which the species under protection remains within a well-defined area. However, speed reductions may be more effective than rerouting to protect species with a broader or variable distribution, or if a change in routing to protect one species risks increasing the impact on another group of animals, then effectively kicking the noise can down the street; this may arise particularly when rerouting vessels from shallow to deeper waters.

### Technological modifications to reduce source levels also reduce impacts to marine mammals

Technological modifications to address ship noise focus primarily on reducing cavitation. Like slowdowns, this reduces the vessel source level, area exposed, and the probability of animals being affected. The efficacy of technological modifications compared to slowdowns depends on how animals are affected by a vessel pass. For example, if a ship is slowed down by 30% from 20 to 14 knots, then its source level drops by a predicted 10 dB. If a similar noise reduction can be achieved by technological improvements without slowing down the vessel, then the maximum sound pressure level received by an animal will be the same as for the slowdown. Behavioral impacts that are mediated by maximum sound pressure level should be, therefore, similar for the slower versus the quieter, fast vessel. However, the exposure duration and acoustic looming show opposite trends. The modified vessel has a shorter exposure duration than the slower unmodified vessel (7.9 versus 12.2 min assuming an ambient noise level of 90 dB re 1 μPa and a closest approach distance of 300 m). Conversely, because acoustic looming depends only on speed, not source level as long as the noise is audible, the modified vessel has the same peak acoustic looming as the unmodified vessel at high speed, whereas the slower unmodified vessel has a lower peak looming (8.9 versus 6.3 dB/min). Consequently, a technologically modified vessel may be less likely to alter the behavior of an animal or mask its hearing due to lower received levels but may still affect an animal if changes in received level as the vessel passes are an important driver for behavioral responses.

How realistic is a 10-dB improvement in source level by technological modifications? Source-level data collected during the ECHO program illustrate that, for the same source depth, the source levels of vessels of the same type and size traveling at the same speed can vary by more than ±10 dB, suggesting that the source levels of the louder vessels can be, in theory, reduced by at least 10 dB (*43*). An important advantage of technological modifications is that they expand the range of speeds at which the vessel can be operated for a given maximum source level. This gives ship operators greater flexibility in reducing noise pollution while balancing economic constraints. However, a potential concern with technologically quietened vessels operated at high speeds is that animals may respond too late to close approaching vessels, thereby increasing the risk of collision.

**Fig. 5.**
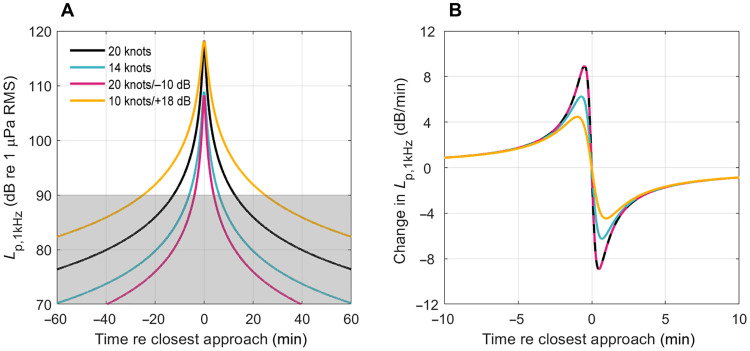
Technological modifications can reduce noise impacts to marine mammals. (**A**) Received levels in the 1-kHz decidecade band (*L*_p,1kHz_) experienced by an animal during the passage of a 295-m container ship with a closest approach distance of 300 m. (**B**) Acoustic looming, i.e., rate of change in *L*_p,1kHz_ experienced by the animal during the passage. Both plots show the vessel traveling at its assumed high speed (20 knots), at a 30% slower speed (14 knots), and at high speed but with technological modifications to reduce source level by 10 dB. The 10-dB noise reduction due to modification has much the same impact on maximum sound pressure level and exposure duration as a 30% slowdown of the unmodified vessel [the 20-knot and 20-knot/−10-dB acoustic looming values are the same and are illustrated using a dashed line in (B)]. However, a noisy vessel can be impactful even when traveling slowly: The orange line in each plot shows the noise level and looming for a vessel traveling at 10 knots but with an 18 dB higher source level (e.g., due to excess cavitation from a damaged or poorly designed propeller). This vessel gives the same maximum received level as the 20-knot reference vessel but elevates the ambient noise (here assumed to be 90 dB re 1 μPa) for twice as long. As acoustic looming depends only on speed, not source level, the slower noisy vessel has a low peak looming. Values and time above ambient are given in table S3.

Some vessels, even when slowed down, can remain loud due to older technology and poor maintenance, resulting in excess cavitation (*31*). To illustrate this, we modeled a vessel traveling at a slow steaming speed (10 knots) but with 18-dB higher source level from excess cavitation compared to our reference vessel at the same speed. For a closest approach distance of 300 m, this noisy vessel gives the same maximum received level as a fast vessel (20 knots) with no technological modifications (118 dB re 1 μPa; Fig. 5A) and twice the exposure duration (50 min; Fig. 5A). Thus, while noisy, slow vessels generate less acoustic looming, they have an elevated impact on animals due to high received levels and longer exposure duration at any given range. Excess cavitation may be caused by a variety of factors, some of which can be mitigated by regular maintenance of the propeller and hull (*19*). Mandatory limits on vessel source levels, irrespective of their speed, would motivate the improvement or elimination of excessively noisy vessels that have repeatedly been shown to be the greatest overall contributors of noise from the global fleet (*28*, *31*).

### Mitigation measures can be combined to further reduce the area exposed to noise

**Fig. 6.**
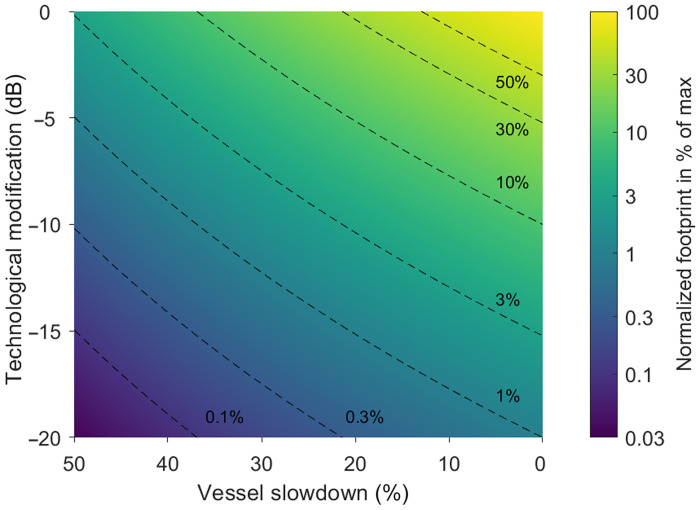
Source-level reduction approaches can be combined to reduce the noise footprint of a cargo vessel. Normalized footprint area reduction (as percent of max) as a function of technological modifications (decibels) and vessel slowdowns (percent), for speeds above the cavitation inception speed. The normalized footprint takes into account the time required for the vessel to pass an area (i.e., a slower vessel ensonifies each point for longer). This results in a slightly lower apparent benefit of speed reduction as compared to the instantaneous footprint (Fig. 2B).

Slowdowns, rerouting, and technological modifications can be combined synergistically to further reduce the areas and number of animals affected by underwater radiated noise. The consequences of different combinations of vessel slowdowns and technological modifications on the acoustic footprint of a vessel assuming spherical spreading can be seen in Fig. 6 (Eq. 8). Here, the top right corner (100%) is equal to the reference condition (unmodified ship traveling at high speed), while the contours show the possible combinations of noise reductions from technological improvements and slowdowns that achieve a given acoustic footprint reduction. For example, a 97% decrease in the normalized footprint (taking time in habitat into consideration) of a vessel could be achieved by a 50% slowdown, a 15-dB technological modification, or a 25% slowdown combined with an 8-dB technological reduction in source level (Fig. 6), so long as the combination of slowdown and technological modifications does not alter the fundamental relationship between radiated noise and speed (Eq. 7). The combination of slowdowns and vessel improvements can result, therefore, in substantial reductions in cumulative noise levels from the global fleet, reducing the areas exposed to vessel noise and the probability of all noise impacts to marine wildlife.

### Recommendations to reduce vessel noise impacts to marine mammals

Global reductions in vessel source levels are required to reduce the impact of shipping activity on marine wildlife. We show that much of the impact of vessel noise can be immediately eliminated by moderate slowdowns of fixed pitch propeller vessels. Reductions in noise effects can also be achieved by increasing the distance between vessels and animals, where possible, and by improving the design of the hull and propeller to reduce cavitation noise. Older noisy vessels that contribute disproportionately to global underwater noise may particularly benefit from refitting or removal from the global fleet. There are, therefore, effective and scalable tools readily available to industry and regulators to directly mitigate the effects of the dominant human noise source in the world’s oceans.

## MATERIALS AND METHODS

The effect of source-level reduction on the amount of habitat exposed to ship noise was modeled on the basis of a reference scenario. This reference scenario comprised the predicted source level of a 295-m-long container ship with fixed pitch propellers, traveling at a high speed of 20 knots, based on the mean length and upper quartile speed of the container ship class recorded by MacGillivray *et al.* (*25*). A vessel with a fixed pitch propeller was selected as this is the propulsion type for the majority of the global fleet (*45*). A container ship was selected as the reference class because (i) they are prevalent within the global commercial fleet (13.2%) (*1*); (ii) transport 90% of general cargo; (iii) generally travel at high speeds, giving them the greatest potential for slowing down; and (iv) have relatively short port times, making them common in open seas (*30*), and, therefore, are likely to encounter marine mammals during transit. We simulated reductions in the source level of the reference vessel through two measures: slower speeds and technological improvements, the latter reducing the source level without affecting speed. The practical effect of a reduced source level was quantified by estimating the associated reduction in ensonified space around the vessel assuming spherical spreading, allowing a quantitative comparison of the efficacy of the two mitigation measures. In addition, we simulated the effect of spatially distancing the reference vessel from animals. All calculations were conducted using custom-written functions in MATLAB 2021a (MathWorks, Natick, MA, USA).

### Area exposed to vessel noise

The area exposed to underwater radiated noise from a cargo vessel (acoustic footprint) was first quantified in terms of its instantaneous acoustic footprint, i.e., the area exposed to underwater radiated noise above the ambient at a point in time. It was assumed that propagation loss followed a simple spherical spreading loss model with distance to the vessel [20log_10_(distance)] given its general applicability (*54*). Spherical spreading is usually the highest level of propagation loss, as sound may attenuate less rapidly with distance in some environments, especially in shallow water. However, everything else being equal, the assumption of spherical spreading leads to an underestimate of the reduction in footprint for a given reduction in source level.

The instantaneous footprint (FP) and swath width (*d*_SW_; see Fig. 2) express how far the potential impact of the radiated noise extends from the ship on the sea surface. These are defined, respectively, as the area around the ship and the surface distance perpendicular from the ships’ direction of travel within which the noise level is above the threshold received level for impacts for a particular animal (*L*_p,min impact_; Eq. 1). This received level is unknown in most cases, but *L*_p,min−impact_ can be assumed to be constant and independent of the speed of the vessel. It is, therefore, possible to calculate the change in swath width (*Q*_sw_) and instantaneous footprint (*Q*_FP_) for a given reduction in source level (Δ*L*_s_) of the ship noise (Eq. 2). First, we define the relationship between *L*_p,min impact_, *L*_s_, and *d*_SW_(*L*_s_) assuming spherical spreading

$$L_{p,\min-\mathrm{impact}}=L_{s}-20\log_{10}\left[\frac{d_{\mathrm{SW}}(L_{s})}{2}\right]\mathrm{dB}$$ (1)

where the notation *d*_SW_(*L*_s_) emphasizes that the swath width is a function of the source level. Equation 1 can be transposed to

$$d_{\mathrm{SW}}(L_{s})=2\;\cdot 10^{\left(\frac{L_{s}-L_{p,\min-\mathrm{impact}}}{20}\right)}$$ (2)

The ratio metric change in swath, *Q*_SW_, corresponding to a reduction in source level of Δ*L*_s_ is

$$Q_{\mathrm{SW}}(\text{Δ}L_{s})=\frac{d_{\mathrm{SW}}(L_{s}-\text{Δ}L_{s})}{d_{\mathrm{SW}}(L_{s})}=10^{\left(\frac{\text{Δ}L_{s}}{20}\right)}$$ (3)

The instantaneous footprint, FP(*L*_s_), as a function of the source level is the area on the sea surface around the vessel, out to a distance of half the swath width [$\frac{d_{\mathrm{SW}}(L_{s})}{2}$; Eq. 4], i.e.

$$\mathrm{FP}(L_{s})=\pi \;\cdot {\left[\frac{d_{\mathrm{SW}}(L_{s})}{2}\right]}^{2}$$ (4)

Combining this with Eq. 3 leads to the ratio metric change in instantaneous footprint, *Q*_FP_, for a given change in source level

$$Q_{\mathrm{FP}}(\text{Δ}L_{s})=\frac{\mathrm{FP}(L_{s}-\text{Δ}L_{s})}{\mathrm{FP}(L_{s})}=\frac{{d_{\mathrm{SW}}}^{2}(L_{s}-\text{Δ}L_{s})}{{d_{\mathrm{SW}}}^{2}(L_{s})}=Q_{\mathrm{SW}}^{2}=10^{\left(\frac{\text{Δ}L_{s}}{10}\right)}$$ (5)

### Change in vessel source levels through speed reduction

The decidecade (also commonly referred to as third octave) source spectrum (*L*_s,ddec_; dB re 1 μPa m) was estimated for the reference scenario using the most recent reference spectrum model (JOMOPANS-ECHO) (*43*). This model has its origins in the model derived by Ross (*32*) and is expressed in Eq. 6, separated into a number of different vessel classes (parameter *C*)

$$L_{s,\mathrm{ddec}}(f,v,l,C)=L_{s0}(f,C)+60\log_{10}\left(\frac{v}{v_{0}}\right)\mathrm{dB}+20\log_{10}\left(\frac{l}{l_{0}}\right)\mathrm{dB}$$ (6)

where *L*_s,ddec_ is the decidecade source level at a given frequency (*f*), *L*_s0_ is the decidecade source level of the vessel class of reference length (*l*_0_) and at the reference speed (*v*_0_), *l* is the length of the actual vessel, and *v* is the actual speed. Equation 6 is only valid for speeds above the cavitation inception speed (*32*, *43*, *46*). The cavitation inception speed for all cargo vessel classes has been suggested to be between 9 and 14 knots (*46*). Modeled slowdowns assumed that the reference vessel traveled with up to a 50% reduction (10 knots) of the high speed (20 knots).

From Eq. 6, the predicted source level changes with speed as

$$\text{Δ}L_{s}(v)=60\log_{10}\left(\frac{v}{v_{0}}\right)\mathrm{dB}$$ (7)

Combining this with Eq. 5 gives a prediction for the change in instantaneous footprint with the ratio metric change in speed $\left(\frac{v}{v_{0}}\right)$

$$Q_{\mathrm{FP}}\left(\frac{v}{v_{0}}\right)=10^{\left(\frac{\text{Δ}L_{s}}{10}\right)}=10^{\frac{60\log_{10}\left(\frac{v}{v_{0}}\right)}{10}}={\left(\frac{v}{v_{0}}\right)}^{6}$$ (8)

See the Supplementary Materials for derivation of the normalized footprint that accounts for the increased time a slower ship spends inside a habitat by normalizing with ship speed and the result that the normalized footprint also decreases with decreasing ship speed.

### Noise impacts to marine mammals from vessels

To quantify changes in the potential noise impacts to marine mammals due to vessel slowdowns, separation distance, and technological modifications, the source level in the 1-kHz decidecade band (*L*_s,1kHz_) was chosen as an example. The shape of the noise spectrum is taken to be unaffected by vessel speed (Eq. 6), which means that the choice of frequency band does not affect the conclusions. The 1-kHz decidecade band was selected because it is audible to all marine mammal functional hearing groups (*7*, *37*).

Noise impact proxies were modeled for the reference vessel (container ship with fixed pitch propellers), assuming that this vessel operated under a range of scenarios. These included the vessel traveling at variable speeds (high and a 20, 30, and 50% slowdown), at different closest-approach distances from an animal (300 and 3000 m), and assuming that the vessel was either slow (10 knots) and with excess cavitation (+18 dB) or fast (20 knots) and had design or technological modifications to reduce source levels (by 10 dB).

As the way underwater radiated noise affects marine mammals remains unclear for many species, three noise impact proxies were chosen as indicative of the potential impact: (i) maximum received level (*L*_p,1kHz_; dB re 1 μPa), (ii) peak acoustic looming (expressed as the rate of change in *L*_p,1kHz_ dB/min), and (iii) exposure duration. Exposure duration was defined as the time (in minutes) during which the received level of vessel noise exceeded the ambient noise level. Each of these impact proxies was derived from the outputs of the following equation (Eq. 9)

$$L_{p,1\mathrm{kHz}}(t)=L_{s,1\mathrm{kHz}}(v)-20\log_{10}\left[\sqrt{c^{2}+{\left(tv\right)}^{2}}\right]$$ (9)

where *L*_p,1kHz_(*t*) is the received level at time *t* [relative to time of closest point of approach (CPA)], *L*_s,1kHz_ is the 1-kHz decidecade source level of a container ship derived from Eq. 6, *v* is the speed, and *c* is the closest perpendicular distance between the vessel track line and the animal on the sea surface. Spherical spreading loss was assumed for the propagation loss regime.

To compare the efficacy of different mitigation strategies, a 1-kHz ambient noise level, *L*_p,amb,1kHz_, of 90 dB re 1 μPa was used as a reference based on the median ambient decidecade sound level in recordings from the Kattegat, Denmark (56° 54.099′N, 11° 38.891′E; Fig. 1C). Ambient noise levels fluctuate depending on local contributions from natural and anthropogenic sources (*4*, *55*), and levels tend to fall between 70 and 110 dB re 1 μPa within frequency bands less than or equal to 1 kHz (*55*, *56*). A lower ambient level will increase the exposure duration but will not affect the maximum received level or acoustic looming unless the ambient noise at the given perpendicular distance is higher than the maximum received level of ship noise.

### Change in exposure duration with speed reduction

We define the exposure duration as the interval during which the vessel noise at a receiving position (*L*_p,1kHz_) is equal to, or greater than, the ambient noise (*L*_p,amb,1kHz_). Defining *t*_e_, as the time (relative to time of CPA) at which *L*_p,1kHz_ is equal to *L*_p,amb,1kHz_, exposure duration is two times *t*_e_ to account for the approach and retreat of the vessel passing the receiving position. Applying Eq. 9, we get

$$L_{p,\mathrm{amb},1\mathrm{kHz}}=L_{s,1\mathrm{kHz}}(v)-10\log_{10}(c^{2}+v^{2}{t_{e}}^{2})$$ (10)

If the exposure duration is long enough, *vt*_e_ >> *c*, then Eq. 10 is approximated by

$$L_{p,\mathrm{amb},1\mathrm{kHz}}\approx L_{s,1\mathrm{kHz}}(v)-20\log_{10}({vt}_{e})$$ (11)

For a baseline vessel speed of *v*_0_, the corresponding value of *t*_e_ is denoted by *t*_e0_ and defined by

$$L_{p,\mathrm{amb},1\mathrm{kHz}}\approx L_{s,1\mathrm{kHz}}(v_{0})-20\log_{10}(v_{0}t_{e0})$$ (12)

Equating Eqs. 11 and 12 gives

$$L_{s,1\mathrm{kHz}}(v)-L_{s,1\mathrm{kHz}}(v_{0})\approx 20\log_{10}({vt}_{e})-20\log_{10}(v_{0}t_{e0})=20\log_{10}\left(\frac{vt_{e}}{v_{0}t_{e0}}\right)$$

Substituting Eq. 7 for the change in source level with speed

$$60\log_{10}\left(\frac{v}{v_{0}}\right)\approx 20\log_{10}\left(\frac{vt_{e}}{v_{0}t_{e0}}\right)$$

into Eq. 8 can be simplified to

$$Q_{\mathrm{ED}}=\frac{t_{e}}{t_{e0}}\approx {\left(\frac{v}{v_{0}}\right)}^{2}$$ (13)

where *Q*_ED_ is the ratio metric change in exposure duration relative to the baseline situation, similar to the definition of *Q*_FP_ above. Thus, a speed reduction of 20% (i.e., *v/v*_0_ = 0.8) drops the exposure duration to 0.64 of its baseline value, a reduction of 36%, independent of the ambient noise level. The approximation in Eq. 13 holds if *t*_e_ is large compared to *c/v*, i.e., if the exposure duration is longer than twice the ratio of the closest approach distance and the speed, which will often be the case.

**Glossary**

• Acoustic looming: This refers to the peak rate of change in vessel received levels over time (decibels per minute), a proxy for presumed sensory cues related to the immediacy of a threat. A fast increasing received level, e.g., due to a vessel approaching at high speeds, is predicted to provoke a stronger or more rapid behavioral response than a slower approaching vessel.

• Exposure duration: This refers to the time over which underwater noise from a passing vessel exceeds the ambient noise level for a particular receiver position. Exposure duration is used as a proxy for the extent to which vessel noise can mask acoustic signals of interest to an animal.

• Instantaneous acoustic footprint: This refers to the surface projection of the volume of water around a ship that is affected by underwater radiated noise at each moment in time. Unit is square kilometers.

• Normalized acoustic footprint: This refers to the instantaneous footprint divided by the speed of the vessel to account for the increased time a slower ship spends inside a habitat. Unit is square kilometers per knot or, in Syst$\overset{`}{e}$me International (SI) units, meters per second.

• Maximum received level: This refers to the maximum received level (*L*_p_; dB re 1 μPa) experienced by an animal during the vessel passage. This is used as a proxy for the severity of a behavioral response, where a higher maximum received level is expected to elicit a more severe behavioral response than a lower received level.

• Swath: This refers to the surface distance perpendicular from the ships’ direction of travel that is exposed to underwater radiated noise from the vessel and thereby a measure of the size of the strip ensonified by the vessel as it moves through the habitat. Unit is meters.

## Supplementary Materials

### This PDF file includes:

Normalizing acoustic footprint with vessel speed Tables S1 to S3
