# Supplementary material for: Small reductions in cargo vessel speed substantially reduce noise impacts to marine mammals
Source: Sci Adv. 2023 Jun 21;9(25):eadf2987. doi: 10.1126/sciadv.adf2987 (PMC10284543; doi:10.1126/sciadv.adf2987)
Supplement: Supplementary file 1 — Normalizing acoustic footprint with vessel speed Tables S1 to S3 [file sciadv.adf2987_sm.pdf]

Science Advances
AAAS

Supplementary Materials for

Small reductions in cargo vessel speed substanstially reduce noise impacts to marine mammals

Charlotte R. Findlay *et al.*

Corresponding author: Charlotte R. Findlay, charlotte.findlay@bio.au.dk

Sci. Adv. **9**, eadf2987 (2023)
DOI: 10.1126/sciadv.adf2987

This PDF file includes:

Normalizing acoustic footprint with vessel speed
Tables S1 to S3

Normalizing acoustic footprint with vessel speed

Two metrics for the area impacted by vessel noise are introduced in the main text: the instantaneous footprint and the normalized footprint (see glossary of terms in the main text). Both of these describe the surface projection of the region around the vessel in which the sound level is elevated above the prevailing ambient noise level due to the ship noise. However, whereas the instantaneous footprint measures the area around the vessel, where ship noise exceeds ambient, the normalized footprint takes into account that a slower vessel spends more time in a habitat. The normalized footprint (FP_{norm}; eqn. S1) is thus the instantaneous footprint (FP_{inst}) normalized by vessel speed (v).

$$\mathrm{FP_{norm}} = \frac{\mathrm{FP_{inst}}}{\mathrm{v}} \tag{S1}$$

FP_{norm} can be expressed in km^2/knot (area exposed per unit of speed), or in SI units, $km \cdot h$. If two ships with identical instantaneous footprints (i.e., having the same source level in the same ambient noise) are compared, one ship 50% slower than the other, then the normalized footprint of the slower vessel is twice that of the fast, because it spends twice as long time in the habitat.

Eqn. 8 of the main text defines how the instantaneous footprint changes as vessel speed changes, assuming that vessel source level depends on speed to the sixth power following MacGillivray and de Jong (*43*):

$$\mathrm{Q_{FP}}\left(\frac{\mathrm{v}}{\mathrm{v_0}}\right) = 10^{\left(\frac{\Delta \mathrm{L_S}}{10}\right)} = 10^{\frac{60\log_{10}\left(\frac{\mathrm{v}}{\mathrm{v_0}}\right)}{10}} = \left(\frac{\mathrm{v}}{\mathrm{v_0}}\right)^6$$

Combining this with eqn. S1 gives the change in the normalized footprint with change in vessel speed (eqn. S2):

$$\mathrm{Q_{norm-footprint}}\left(\frac{\mathrm{v}}{\mathrm{v_0}}\right) = \frac{\frac{\mathrm{FP_{inst}(v)}}{\mathrm{v}}}{\frac{\mathrm{FP_{inst}(v_0)}}{\mathrm{v_0}}} = \mathrm{Q_{inst-footprint}} \cdot \left(\frac{\mathrm{v}}{\mathrm{v_0}}\right)^{-1} = \left(\frac{\mathrm{v}}{\mathrm{v_0}}\right)^5 \tag{S2}$$

Thus, the normalized footprint also decreases as speed decreases but by a lower power law of five.

% Slowdown	Speed (kn)	$L_{s,broadband}$ (dB re 1 µPa m)	$L_{s,1kHz}$ (dB re 1 µPa m)	LF Cetacean	HF Cetacean	VHF Cetacean	PCW	OCW	ΔL_s (dB)	FP_{inst} (%) Spherical	FP_{inst} (%) Cylindrical
0	20	193	168	168	139	130	162	163	0	100	100
10	18	190	165	165	136	127	159	160	3	50	25
20	16	187	162	162	133	124	156	157	6	25	6
30	14	183	158	158	129	121	153	154	10	10	1
40	12	179	154	154	125	117	149	150	14	4	0.16
50	10	175	150	150	121	112	144	145	18	1.5	0.03

Table S1. Broadband ($L_{s,broadband}$) and 1 kHz ($L_{s,1kHz}$) decidecade source levels of a 295 m fixed pitch container ship travelling at a reference speed of 20 knots (kn) and at various slower speeds. Source levels are also shown weighted for marine mammal functional hearing groups (LF – Low-Frequency cetaceans, HF – High-Frequency cetaceans, VHF – Very High-Frequency cetaceans, PCW – Phocid Carnivores in Water, and OCW – Other Carnivores in Water)(*37*). The last three columns give the decibel source level reduction (ΔL_s) attained by each slowdown scenario, and the corresponding instantaneous footprint (FP_{inst}) area, i.e., the percentage area exposed to vessel noise assuming either spherical or cylindrical spreading loss models.

CPA (m)	% Slowdown	Maximum $L_{p,1kHz}$ (dB re 1 μPa RMS)	Exposure Duration (minutes and %)	Peak Acoustic Looming (dB/min)
300	0	118	25 (0%)	8.9
	20	112	16 (36%)	7.1
	50	100	6 (76%)	4.5
3000	0	98	23 (0%)	0.9
	20	92	10.4 (54.8%)	0.7
	50	80	NA (100%)	0.4

Table S2. Noise impact proxies for a vessel passing an animal with a closest point of approach (CPA) distance of 300 m and 3000 m. The vessel is a 295 m container ship operating at 20 knots (the reference scenario) and various slower speeds. The impact proxies are: (i) the 1 kHz decidecade received level at maximum exposure; $L_{p,1kHz}$, (ii) the exposure duration, assuming an ambient level ($L_{p,amb,1kHz}$) of 90 dB re μPa, and (iii) the peak acoustic looming (maximum rate of change in sound level). The exposure duration is also expressed as a percentage reduction of the exposure duration compared to the reference speed.

**Speed (kn) / Source Difference (dB)**	**$L_{s,1\ kHz}$ (dB re 1 µPa m)**	**Maximum $L_{p,1kHz}$ (dB re 1 µPa RMS)**	**Exposure Duration (minutes)**	**Peak Acoustic Looming (dB/min)**
20 / 0	168	118	25	8.9
14 / 0	165	109	12.2	6.3
20 / -10	158	108	7.9	8.9
10 / + 18	168	118	50	4.7

Table S3. Estimated source level and impact proxies for various vessel speed and noise reduction combinations. The vessel is a 295 m container ship passing with a CPA of 300 m from an animal. The reference scenario on row 1 is an unmodified ship travelling at 20 knots. Additional rows in the table show the effect of (i) a 30% slowdown (to 14 kn), (ii) technological modifications to reduce source levels by 10 dB at 20 kn, and (iii) a slow (10 kn) but loud (+18 dB) vessel. The impact proxies are the 1 kHz decidecade received level at maximum exposure ($L_{p,1kHz}$), exposure duration, and peak acoustic looming (maximum rate of change in sound level)
